# University of Buffalo 1846—1909

**Published:** 1909-07

**Authors:** 


BUFFALO MEDICAL JOURNAL.

A Monthly Review of Medicine and Surgery.

*EDITOR*

WILLIAM WARREN POTTER, M. D.

All communications, whether of a literary or business nature, books for review and exchanges, should be addressed to the editor, 238 DELAWARE AVENUE, BUFFALO, N.Y.

VOL. LXIV. JULY, 1909. No. 12

### University of Buffalo 1846—1909

THE sixty-third annual commencement of the department of medicine, the twenty-second annual commencement of the department of pharmacy, the twenty-first annual commencement of the department of law and the sixteenth annual commencement of the department of dentistry of the University of Buffalo took place at the Teck Theater, Friday, May 28, 1909, at 11 A. M. The preliminary announcement of the exercises during commencement week was published in the June issue of the JOURNAL. The magazine, however, went to press too early to give the full proceedings, hence we publish them in this edition, thus making the historic chain complete:

THE MEDICAL ALUMNI ASSOCIATION.

This association, organized in 1874, held its Thirty-Fourth Annual Meeting, May 25, 26, 27, and 28, 1909. The program for Tuesday evening, May 25, was as follows:

8.00 P. M.—The classes of '49, '59, '69, '79, '89, '99 met in the college building for organisation and registration.

8.30 P. M.—The association transacted routine business and elected officers for the ensuing year.

9.00 P. M.—President Albert T. Lytle, '93, delivered the annual address. Professor Frederick C. Busch, '97, followed with an address entitled, "Modern Experimentation in Relation to Medicine and Surgery."

In his discourse Dr. Busch described his experiments in transplanting adrenals, from one animal to another, obtaining a functional survival of the graft.

11.00 P. M.—A supper and smoker in the college library were served by the association.

The election of officers resulted as follows: president, H. J. Nichols, Bradford, Pa.; vice-presidents, Marshall Clinton, Buffalo; Charles L. Preish, Lockport; Miss N. Victoria Chappell, Buffalo; E. B. Horton, Niagara Falls, and E. A. Forsyth, Buffalo; treasurer, Herman K. DeGroat of Buffalo; secretary,

Franklin W. Barrows of Buffalo, and chairman of the executive committee, Dr. Frederick C. Busch, Dr. Albert T. Lytle, the retiring president, was made a trustee.

Beginning Wednesday morning, May 26, an exhibit of pathologic specimens, preparations, and photographs were arranged in the College Museum under the direction of Professor H. U. Williams, these remaining on view during the entire meeting.

A series of clinics at the Buffalo General Hospital and at the Buffalo Hospital of the Sisters of Charity, were held during Wednesday and Thursday, under the following schedule: Wednesday, 9 to 11 A. M.—Gynecology, Professors Mann and King. 11 to 1 P. M.—Neurology, Professors Putnam and Krauss. 1.30 P. M.—Luncheon served in the college library by the Association. 2 to 5 P. M.—The staff of the Buffalo Hospital of the Sisters of Charity, presented a series of unusual and interesting cases from the various departments, by Professors Hanley, Meyer, Smith, Buswell, Clinton, Dooley, Kaiser, Mooney, Howell.

On Thursday, May 27, clinics continued at the Buffalo General Hospital as follows: 9 to 11 A. M.—Pediatrics, Professors Snow and Sherman. 11 to 1 P. M.—Surgery, Professors Park and Phelps. 2 to 4 P. M.—Medicine, Professors Stockton, Cary and Hopkins. 4 to 6 P. M.—Otology, Laryngology, Professors Hinkel and Cott. 5 to 6 P. M.—Ophthalmology, Professors Howe and Burrows. 8.00 P. M.—Fraternity night.

The program for Friday, May 28, was as follows: 11.00 A. M.—Sixty-fourth annual commencement exercises of the University of Buffalo, including the Departments of Medicine, Law, Pharmacy and Dentistry, took place at the Teck Theatre. Professor Adelbert Moot, LL. B., delivered the address. 1.00 P. M.—The faculty of the medical department received the graduates, their friends and the alumni at the University Club, where luncheon was served.

### Chancellor Norton.

Professor Moot, in the beginning of his address made announcement that Vice-Chancellor Norton had been created Chancellor that morning, by unanimous vote of the Council. Nothing in the form of an exodium could have pleased the audience more, its gratification being testified in generous applause. Chancellor Norton previously announced that the $50,000 necessary to secure the Main street site for the Greater University had been subscribed.

### Conferring of Degrees.

Chancellor Norton conferred degrees on one hundred and seven candidates, the graduates being distributed among the four departments as follows: medicine, 34; pharmacy, 28; law, 26, and dentistry, 19. Those receiving medical degrees were Horace James Beel, Medina, N. Y.; Frank Edwin Brundage, Ph.B., 572 W. Ferry St., Buffalo, N. Y.; Paul Curtis Campbell, 365 Walnut St., Lockport, N. Y.; Anthony J. Cetola, 65 Front Ave., Buffalo, N. Y.; Edward Daniels Clark, 48 W. Oakwood Place, Buffalo, N. Y.; Julius Cohen, 180 Hodge Ave., Buffalo, N. Y.; David Gordon Cooper, Lyndonville, N. Y.; Samuel S. Creighton, 448 Elk St., Buffalo, N. Y.; Charles Denton Cromwell, Peoria, N. Y.; Warren Z. Dell, 99 Harvard Place, Buffalo, N. Y.; Howard Cousins Fairbanks, Hermon, N. Y.; Raymond Cornelius Fess, Bowmansville, N. Y.; Roy Crowder Fisher, 42 Lawrence Place, Buffalo, N. Y.; Gilbert de Leverance Forbes, Kendall, N. Y.; William Francis Gallivan, 125 Walter St., Buffalo, N. Y.; Joseph P. Gimbrone, 247 Front Ave., Buffalo, N. Y.; James V. Gowans, Attica, N. Y.; John Stephen Herlihy, Akron, N. Y.; Charles Gordon Heyd, B. A., 418 Sherbourne St., Toronto, Ont.; Allen Wheeler Holmes, West Falls, N. Y.; Joseph Edward Hurley, 10 Phelps Ave., Rochester, N. Y.; Benjamin Jacobson, 159 Cedar St., Buffalo, N. Y.; Frank Christian Kleckner, 342 Monroe St., Buffalo, N. Y.; Charles George Lenhart, Lockport, N. Y.; Harry Eugene Lyons, 3050 Peach St., Erie, Pa.; George Patrick McCarthy, Friendship, N. Y.; Herman Frank May, 641 Elm St., Buffalo, N. Y.; Rudolf Christian Miller, Ph. G., 596 Broadway, Buffalo, N. Y.; Eugene William Rother, 444 Pearl St., Buffalo, N. Y.; Bernard Francis Schreiner, 121 Ash St., Buffalo, N. Y.; Archibald Wilson Thomson, 22 William St., New York City; Frank Hamilton Towne, Silver Creek, N. Y.; William Joseph Tracy, 301 Canisteo St., Hornell, N. Y.; Frank Gebhard Walz, 159 Howard St., Buffalo, N. Y.

### Incidents.

The Medical Department offers no prizes, but first, second and third honors went to Charles Gordon Heyd, A. B. Frank, Gebhard Walz and Rudolph Christian Miller, Ph. G.

Dr. Lucien Howe, according to his usual custom, entertained the medical graduating class of 1909, at the University Club, Wednesday evening, about forty attending the dinner.

Renewing old friendships, swapping reminiscences, and singing college songs, the class of 1899, of the Medical Department at the University of Buffalo, held a banquet to celebrate its 10th anniversary of graduation, in the Lafayette Hotel, Wednesday evening, May 26. Twenty members of the class put in an appearance. The greatest enthusiasm was evinced when mention was made, in one of the speeches, of the near approach of the greater "Queen City" University. Dr. Francis F. O'Gorman was toastmaster. The speakers were Dr. George Staniland, Dr. A. F. Zittle, Dr. Julius F. Riester, Dr. Louis Beyer, Dr. Ira Livermore of Shelby and Dr. Fred Eckerson of Gowanda.

Something more should be said of that brilliant young Chancellor, Mr. Charles P. Norton, who has succeeded in making possible the "Greater" University of Buffalo. He has literally wrung victory from defeat, and we cannot present the case in better form than did the *Evening News* in its editorial of May 29, 1909, from which we quote.

Chancellor Norton's Success.

Charles P. Norton has been Vice-Chancellor of the University of Buffalo during a period devoted to obtaining a site in preparation for a department of arts and letters on the University foundation. Last summer he was given an option of lands at the city line on Main street with a year in which to collect the price and take the property. The deal was made with the county after discussions as exasperating for their stupidity on the part of some opponents of it as they were needlessly prolonged. It was an almost heartbreaking battle to get through the Board of Supervisors but it was won by the side devoted to liberal ideas of education and Mr. Norton at once began another campaign for the funds to make the chance of the University good.

It is announced that Mr. Norton has succeeded in his quest. His persistence and pertinacity, his insensibility to rebuff, his ingenuity of argument and appeal or, to put it the other way, his splendid enthusiasm, his determination to win, his disinterestedness and his business skill made him invincible. He has declined to take no for an answer and never failed to return to the charge until he had gained some success from his assault.

It was with a conviction that he was called to a high and noble task that Mr. Norton undertook the work of raising funds for the University. It was in the very midst of the panic that he had to make his start. His burden was all the harder from the circumstances of industry at the time but nothing could stop him or lessen his activity in the sacred cause, as he looks at it. He has kept the faith and fought a good fight and won a grand victory. A man could not ask a finer monument of his interest in a community than such an addition to the University as he has done so much to establish.

Charles Gordon Heyd, Buffalo, entered the competitive examination in New York for appointment to the Post-Graduate Hospital. There were 31 candidates, representing Harvard, Yale, McGill, Cornell, Ann Arbor, Johns Hopkins, Columbia, Pennsylvania and others. Heyd stood highest and received first appointment.

Dr. Joseph P. Brennan, Buffalo, graduate of class of 1908, Wm. F. Gallivan, Buffalo, class of 1909, entered the competitive examination for appointments in Bellevue Hospital, proper.

There were 22 candidates, representing Cornell, Columbia, Vanderbilt (Nashville, Tenn.), McGill. Brennan passed highest and received senior appointment for two years. Gallivan stood seventh and received appointment for one year.

Hospital appointments were distributed as follows: Horace James Beel, Butterworth Hospital, Grand Rapids, Mich.; Paul Curtis Campbell, Emergency Hospital, Buffalo; Edward Daniels Clark, Buffalo General Hospital; Julius Cohen, Buffalo Hospital of the Sisters of Charity; David Gordon Cooper, Buffalo General Hospital; Samuel S. Creighton, Buffalo General Hospital; Charles D. Cromwell, Buffalo General Hospital; Warren Z. Dell, Erie County Hospital, Buffalo; Howard C. Fairbanks, Emergency Hospital, Buffalo; Raymond C. Fess, German Deaconess Hospital, Buffalo; Gilbert DeL. Forbes, Rochester City Hospital; Wm. F. Gallivan, Bellevue Hospital, New York City; James V. Gowans, Buffalo Hospital of the Sisters of Charity; John S. Herlihy, Buffalo Hospital of the Sisters of Charity; Charles G. Heyd, B. A., New York Post-Graduate Hospital, New York City; Allen W. Holmes, Buffalo General Hospital; Joseph E. Hurley, St. Mary's Hospital, Rochester, N. Y.; Charles G. Lenhart, Erie County Hospital, Buffalo; Harry E. Lyons, St. Vincent's Hospital, Erie, Pa.; George P. McCarthy, Emergency Hospital, Buffalo; Herman F. May, Erie County Hospital, Buffalo; Rudolf C. Miller, Ph. G., Buffalo General Hospital; Eugene W. Rother, Buffalo General Hospital; Bernard F. Schreiner, Buffalo General Hospital; Archibald W. Thomson, Buffalo Hospital of the Sisters of Charity; Frank H. Towne, Buffalo General Hospital; William J. Tracy, Buffalo Hospital of the Sisters of Charity; Frank G. Walz, Buffalo Hospital of the Sisters of Charity. Thirty-four graduates; twenty-nine hospital appointments.

---

In a warning against the great summer pest and menace—mosquitoes—the Department of Health of Philadelphia says: "Mosquitoes, as a rule, deposit their eggs in stagnant water, which hatch out with great rapidity in the form of small animals called 'wrigglers,' named from the manner in which they propel themselves through the water, which, in a few days, become full-fledged mosquitoes. A sufficient number of mosquitoes to annoy an entire neighborhood can be developed from a very small quantity of water. A neglected tomato can containing water is sufficient for this purpose; therefore, watch, remove, empty or tightly cover with cheesecloth all barrels, boxes, tin cans, choked roof gutters, sewer traps or any receptacle where water can lie for a period of five days."

At a meeting of the Regents of the University of the State of New York, held at Albany, June 17, 1909, appointments were made as usual as follows:

State Board of Medical Examiners—Dr. William S. Ely, Rochester, whose term expires on July 31, reappointed for a term of three years; new appointees, Dr. William H. Park, New York, and Dr. Willis G. Macdonald, Albany.

---

An extensive plan to improve higher education is set forth in the announcement of the Higher Education Association which has filed articles of incorporation at Albany with a capital of $300,000. The incorporators include President John H. Finley of the College of the City of New York, Edwin E. Slosson, editor of the *Independent*, and George B. Cortelyou, who was at one time principal of preparatory schools in New York.

The purpose of the organisation, as set forth in its articles of incorporation, is "to improve higher education throughout the United States, and in particular the internal and external conditions of the American college, by furnishing an agency and funds whereby a careful study can be made and improvement can be brought about in the institutions of higher learning."

The plans contemplate reorganisation of the financial departments of colleges, with development of an internal cost accounting system.

---

Governor Hughes in the latter days of May, vetoed the C. F. Brown bill, amending generally the public-health law relative to pharmacy. Concerning his action the Governor said:

The present bill provides for a new board of pharmacy, to consist of nine examiners, to be designated by the regents of the state university. While, so far as examinations and licenses are concerned, it might be proper to have the board constituted in this way, the advisability of investing such a board of examiners with the broad powers provided for in the bill is open to serious question.

It is provided, however, that the first examiners are to be designated from the members of the present board, and that in appointing their successors the appointment by the regents shall be made from the names of six candidates to be submitted to the regents by the pharmaceutical association. Thus the powers given by the bill are to be exercised by those designated in this manner by a private organisation. I do not believe in this policy.

---

To warn people of the dangers of flies, and to show them how to get rid of the pests, the Chicago Health Department has issued a bulletin, in which the pesky nuisances are called all sorts of bad names. "Flies are the dirtiest and filthiest of vermin," the bulletin says. "They are born in filth, live in filth and carry filth around with them. Millions of death-dealing germs cling to them, only to be scattered upon those whom they touch. Now is the time to build your lines of defence. Prepare to fight them as you would wild beasts seeking your life." A good fly poison, not dangerous to human life, the bulletin adds, is a solution of bichromate of potash, one dram dissolved in two ounces of water, and sweetened with a little sugar. Put some in shallow dishes and place throughout the house. Another is cobalt chloride, one dram dissolved in three ounces of water, placed in shallow dishes as above. To clear rooms in which there are large numbers of flies burn pyrethrum powder or blow black flag into the air of the room. These do not kill the flies; they are merely stunned and fall to the floor. They must then be gathered up and destroyed.

---

The United States government will be represented at five important medical conferences in Europe this summer by Medical Director J. C. Wise and Surgeon Frank L. Pleadwell, of the navy, both of whom sailed for Europe from New York on June 19, 1909.

The International Antitubercular Congress at Stockholm, July 8 to 10, will be attended by Medical Director Wise, who afterward will go to Bergen to be present at the International Scientific Congress on Leprosy, August 16 to 19, and then visit Budapest for the meeting of the International Medical Congress, which meets there August 29 to September 4.

Surgeon Pleadwell will go first to Paris for the second international conference for the revision of nomenclature of diseases and causes of death, July 1 to 3, and then to London to be present at the Twelfth International Congress on Alcoholism to be held July 18 to 24. Both of the American representatives will present important papers at some of the meetings.

---

The Buffalo day camp for tuberculosis patients was opened June 1, 1909. It is located on Main street immediately north of the Lackawanna railway tracks, and has accommodations for sixty patients. This day camp is an important feature of the systematic warfare which Buffalo is making against consumption.

---

As a result of an amendment made to the public health law by the last Legislature, physicians will have more work to perform. This provides that when a patient dies the physician must within twenty-four hours deliver to the local registrar a certificate of the death and the probable cause. The amendment transfers the responsibility from the undertaker to the physician, relieving the undertaker of the necessity of calling on the physician for the necessary data.

Under the new law, in case an inquest is required, the Coroner or the Coroner's physician must fill out the certificate, and if no inquest is required and no physician has been in attendance, the certificate shall be filled out by some reputable persons known to the official issuing the burial permit, and the person thus acting must make an affidavit to the facts set forth in the death certificate The old law required an undertaker to file the physician's certificate before securing a burial permit. The amendment permits the State Health Department to deal directly with the physicians.

Another change requires that the registration of a birth must be made within thirty-six hours to the local health authorities, the object of this provision being to enable the Health Department to arrest at once any disease that may prove to be serious if not attended to.

---

A NEW anesthetic which, it is said, will prove a great boon to the medical profession, is reported by the American Consul at Bucharest, Rumania, to have been discovered there by an eminent physician. It is composed of strychnine and storeine, and is said to have practically the same effect as cocaine, except that it can be used in major operations and is not applied locally. The drug is infused into the system by injection and causes the patient to lose all sensation, but does not rob him of consciousness. For operations below the waist, the anesthetic is injected at the base of the spine, and for operations above the waist it is infused into the spine, between the shoulders.

The consul reports to the department that the drug has proved successful in many cases in Rumania, and that its discoverer is on his way to London to have it tested by the medical authorities.

---

MR. WILLIAM JAY SCHIEFFELIN makes the interesting suggestion, according to *The Tribune,* that a prohibitory duty be placed on imported cocaine, in order to restrict the irregular and improper sale of the drug in this country. He calls attention to the extent to which unscrupulous druggists and physicians are dealing in and employing cocaine, which they obtain by importation from England and Germany. By this means they evade the New York law, which compels the manufacturer and wholesaler to keep a record of the persons to whom they sell the drug. If a prohibitory duty would restrict the abuse of cocaine, much could assuredly be said in its favor, but the complications of our state system, with its various laws suggest certain doubts. Mr. Schieffelin's efforts to get at the abuse are praiseworthy, and his suggestion of a prohibitory duty deserves consideration.

